# The Place of FDG PET/CT in Renal Cell Carcinoma: Value and Limitations

**DOI:** 10.3389/fonc.2016.00201

**Published:** 2016-09-06

**Authors:** Yiyan Liu

**Affiliations:** ^1^Nuclear Medicine Service, Department of Radiology, New Jersey Medical School, Rutgers University, Newark, NJ, USA

**Keywords:** renal cell carcinoma, FDG PET/CT, staging, restaging, tyrosine kinase inhibitors

## Abstract

Unlike for most other malignancies, application of FDG PET/CT is limited for renal cell carcinoma (RCC), mainly due to physiological excretion of 18F-fluoro-2-deoxy-2-d-glucose (FDG) from the kidneys, which decreases contrast between renal lesions and normal tissue, and may obscure or mask the lesions of the kidneys. Published clinical observations were discordant regarding the role of FDG PET/CT in diagnosing and staging RCC, and FDG PET/CT is not recommended for this purpose based on current national and international guidelines. However, quantitative FDG PET/CT imaging may facilitate the prediction of the degree of tumor differentiation and allows for prognosis of the disease. FDG PET/CT has potency as an imaging biomarker to provide useful information about patient’s survival. FDG PET/CT can be effectively used for postoperative surveillance and restaging with high sensitivity, specificity, and accuracy, as early diagnosis of recurrent/metastatic disease can drastically affect therapeutic decision and alter outcome of patients. FDG uptake is helpful for differentiating benign or bland emboli from tumor thrombosis in RCC patients. FDG PET/CT also has higher sensitivity and accuracy when compared with bone scan to detect RCC metastasis to the bone. FDG PET/CT can play a strong clinical role in the management of recurrent and metastatic RCC. In monitoring the efficacy of new target therapy such as tyrosine kinase inhibitors (TKIs) treatment for advanced RCC, FDG PET/CT has been increasingly used to assess the therapeutic efficacy, and change in FDG uptake is a strong indicator of biological response to TKI.

## Introduction

Renal cell carcinoma (RCC) is the most common solid tumor of the kidneys, accounting for 3% of all malignancies and representing the seventh leading cause of cancer. The most common histological subtype of RCC is clear cell RCC, followed by papillary carcinoma. Standard imaging evaluation for the characterization of primary renal tumor includes ultrasound, CT, and MRI. Cross-sectional imaging, especially contrast CT, is a primary imaging modality for tumor detection and diagnosis, and its increasing use has led to an increased diagnosis of RCC. Surgical resection through either partial or radical nephrectomy remains the mainstay of treatment for the localized disease.

Positron emission tomography (PET) has emerged as one of the most important imaging modality in staging, restaging, detecting recurrence and/or metastasis, and monitoring therapeutic response in most malignant diseases (1, 2). In PET, a trace amount of a radioactive compound is administered, and the resultant images are obtained from three-dimensional spatial reconstructions. The intensity of the imaging signal is proportional to the amount of tracer and, therefore, is potentially semiquantitative (3). Whereas conventional imaging techniques can provide information on anatomic abnormalities, PET imaging relies on both molecular biology and *in vivo* imaging to provide information about the preceding changes in metabolism and function, including glucose metabolism, cell proliferation, cell membrane metabolism, or receptor expression. Furthermore, integrated PET/CT units allow correct co-registration and fused imaging of anatomical and functional data. The integration of CT imaging with PET has been demonstrated to significantly decrease false positive results and improve accuracy of the PET study (4–6).

18F-fluoro-2-deoxy-2-d-glucose (FDG), a non-physiological radiotracer with a chemical structure similar to that of naturally occurring glucose, is most commonly used in PET imaging. FDG enters cells through the same membrane glucose transporter proteins utilized by glucose, which are commonly overexpressed in cancer cells (7, 8). FDG imaging relies upon Warburg’s observation that increased glycolysis generated adenosine triphosphate is required to meet the metabolic demands of rapidly dividing tumor cells. Membrane glucose transporters, mainly GLUT-1, actively transport FDG into the cell, where hexokinase then converts it into FDG-6-phosphate. As FDG-6-phosphate is not a substrate for further steps in glycolysis, it is trapped in the cell and accumulates correspondingly to the cell’s glucose metabolic activity. FDG accumulation rate is semiquantitatively measured by the standardized uptake value (SUV). Malignant cells exhibit increased FDG accumulation due to increased membrane transporters, increased intracellular hexokinase, and low glucose-6-phosphatase (8).

Unlike for most other malignancies, application of FDG PET/CT is limited for RCC, mainly due to physiological excretion of FDG from the kidneys, which decreases contrast between renal lesions and normal tissue, and may obscure or mask the lesions of the kidneys. However, published clinical observations were discordant. In the era of PET/CT in oncology, clarification and validation of FDG PET/CT for RCC is of great significance for urologists, oncologists, and radiologists. This review presents the studies regarding the FDG PET/CT for RCC. The role of FDG PET/CT is discussed based on the critical, non-structured review of the literature.

## FDG PET/CT for Primary RCC

Many early clinical observations showed unfavorable results about the role of FDG PET/CT for detection and characterization of lesions of the kidney, with pooled sensitivity of 50–60% (9). Even forced diuresis coupled with parenteral hydration could not improve the sensitivity (10). In Miyakita’s study (11), 19 consecutive patients with RCC were imaged using FDG PET preoperatively, the results of which were then compared with the histology obtained after radical surgery. Increased FDG uptake was found in only in 6 out of the 19 patients (31.5%) while immunohistochemistry of GLUT-1 in RCC produced varying results; there was no correlation of GLUT-1 immunoreactivity and FDG PET positivity. Aide et al. prospectively compared the efficiency of FDG PET with diagnostic CT in the characterization and primary staging of 35 suspicious renal masses (12). A high rate of false negative results was reported with FDG PET, leading to 47% sensitivity, 80% specificity, and 51% accuracy; all lower than those of CT. The author concluded that, in the characterization of renal masses, FDG PET imaging does not offer any additional advantages compared with CT. In another retrospective study of 66 patients with known RCC by Kang et al. (13), the accuracies of FDG PET and conventional imaging modalities were also compared. FDG PET exhibited a sensitivity of 60% and specificity of 100% for primary RCC tumors, while abdominal CT demonstrated 91.7% sensitivity and 100% specificity. Ozulker et al. evaluated the efficacy of FDG PET/CT in the detection of RCC in patients with indeterminate renal masses detected by conventional imaging from 18 patients (14). All patients underwent nephrectomy or surgical resection of the renal mass, and the final diagnoses were based on histopathology. Fifteen patients had RCC, and three renal tumors were benign. FDG PET/CT accurately detected seven malignant lesions and false negative results in eight patients. FDG PET/CT yielded true negatives in two cases of renal cortical cyst and false positive in one case with oncocytoma. For primary RCC tumors, PET showed 46.6% sensitivity, 66.6% specificity, and 50% accuracy. The median size of visualized tumors was greater than that of non-visualized tumors, and the average Fuhrman grade of the patients with FDG-positive malignant lesions were higher than that of the patients with FDG-negative lesions. There was no significant change in average and maximum SUVs between early and delayed imaging for malignant tumors.

However, some clinical observations demonstrated favorable results regarding the role FDG PET/CT in RCC and showed high FDG avidity in the majority of RCC lesions. In a study by Kumar et al. (15), FDG PET was performed in 28 solid renal masses visualized by CT/MRI. Of the lesions, FDG PET was accurately able to depict 23 out of 27 (85%) malignant renal masses. Of the 10 primary renal tumors (9 malignant, 1 benign), FDG PET yielded 8 out of 9 true positive results (89%), 1 true negative, and 1 false negative. In addition to characterization of the lesions, FDG PET also contributed to primary staging, altering management in 3 out of 10 patients (30%). Of metastatic renal tumors, FDG PET was positive in 15 out of 18 (83%). There was no significant difference in SUVs between primary and metastatic renal masses. Nakhoda et al. evaluated the sensitivity of FDG PET/CT to detect different renal lesions (16). Fifteen out of 18 RCC were detectable by PET, whereas all renal lymphomas and metastases were detectable. None of the metabolic parameters were statistically significant between RCC and renal lymphoma. However, all metabolic parameters were statistically and significantly greater for renal metastases compared with RCC and renal lymphoma, and for clear cell RCC compared with papillary RCC. In addition to a sensitivity of 88% for detection of solid malignant renal lesions in patients with known renal malignancy, FDG PET/CT also reveals differences in metabolic activity based on histopathological type.

Recently, Takahashi et al. retrospectively analyzed FDG PET/CT findings in 98 lesions from 93 patients who had partial or radical nephrectomy after imaging (17). The SUVs of high-grade clear cell RCC were significantly higher when compared with that of the control benign lesions and low-grade tumors. An optimal SUV cutoff value of 3.0 had 89% sensitivity and 87% specificity in differentiating between high-grade and low-grade clear cell RCCs. Multiple regression analysis demonstrated that a high-grade clear cell RCC was the most significant predictor of SUV.

Overall, the results were heterogeneous. Although FDG PET/CT may be helpful in the characterization and detection of primary renal tumors, it has low negative predictive value. In addition, it seems that FDG PET/CT does not have significant advantage in diagnosis and staging of RCC compared with the diagnostic CT.

## Predictive Role of FDG PET/CT in Prognosis

Metabolic quantitation by SUV measurement on FDG PET/CT may play a role in the evaluation of biological behavior of lesion and prediction of patient’s prognosis. Namura et al. evaluated the impact of the maximum SUV (SUV_max_) from FDG PET/CT on survival in 26 patients with advanced RCC (18). High SUV_max_ in patients with RCC correlated with poor prognosis, as there was a statistically significant difference in the survival between patients with SUV_max_ equal or greater than the mean of SUV_max_, 8.8 and patients with SUV_max_ less than 8.8. The authors revealed that the SUV_max_ might have a role as a novel biomarker in prognosticating the survival time of patients with advanced RCC by multivariate analyses with standard risk factors or risk classifications. In another study by Ferda et al. (19), 60 RCC patients had follow-ups for development of the disease 12 months after FDG PET/CT. The highest FDG accumulation was seen in the tumor of the highest grade, and the highest mortality was found for tumors exceeding SUV_max_ of 10. Lee et al. investigated the relationship between the SUV_max_ of primary RCC with and without metastatic lesions in 23 patients (20). The median SUV_max_ of primary RCC of the 16 patients without metastasis was 2.6 (range of 1.1–5.6) while that of the patients with metastasis was 5.0 (range of 2.9–7.6). The SUV_max_ of the primary RCC with metastasis (5.3 ± 1.7) was significantly higher than those without metastasis (2.9 ± 1.0). Thus, one of the roles of FDG PET/CT in the initial evaluation of a patient with RCC may be in predicting extrarenal disease, as patients who have primary RCC with high SUV_max_ are suggested to have a likelihood of metastasis.

Based on the limited data, quantitative FDG PET/CT imaging may facilitate the prediction of the degree of tumor differentiation and allow for prognosis of the disease. FDG PET/CT may be an effective imaging biomarker to provide useful information about patient’s survival. However, more studies are needed to justify these preliminary findings.

## FDG PET/CT for Restaging RCC

Metastatic RCC is one of the most lethal urologic cancers. Up to one-third of patients with newly diagnosed RCC have metastatic diseases (21). Even after nephrectomy of a locally confined disease, more than 30% of the patients develop metastases, most commonly to the lung, bone, skin, liver, and brain (21). Effective staging of RCC, therefore, is crucial for the management of patients.

Although the role of FDG PET/CT in diagnosing RCC is conflicting, it has been more effective in the detection of metastatic disease, thus affecting therapeutic decisions. Obviously, size of the lesions has been shown to be a significant factor affecting sensitivity of PET/CT. Majhail et al. evaluated the performance of FDG PET in detecting metastatic lesions in 24 patients with histologically proven RCC and suspected distant metastases based on conventional anatomic imaging (22). Histologically documented distant metastases were present in 33 sites. Overall sensitivity, specificity, and positive predictive value of FDG PET for the detection of distant metastases from RCC was 63.6% (21 out of 33), 100% (3 out of 3), and 100% (21 out of 21), respectively. The mean size of distant metastases in patients with true positive FDG PET was 2.2 cm (95% CI, 1.7–2.6 cm) compared with 1.0 cm in patients with false negative FDG PET.

18F-fluoro-2-deoxy-2-d-glucose (FDG) PET seems useful for postoperative surveillance in patients with RCC. It can detect recurrence in the surgical site. Nakatani et al. evaluated the surveillance role of FDG PET in 23 postoperative patients with RCC (23). Histological final diagnosis of at least 6 months clinical follow-up was used to confirm diagnostic accuracy of visually interpreted PET. FDG PET was demonstrated to have 81% sensitivity, 71% specificity, and 79% diagnostic accuracy. PET was able to accurately detect local recurrence and metastases to the peritoneum, bone, muscle, and adrenal gland in all cases. In six cases (21%), additional information was obtained from scans, ultimately affecting the course of therapeutic management in three cases (11%). The cumulative survival rate over 5 years in the PET-positive was 46%, whereas that of the PET-negative group was 83%. Kumar et al. assessed 103 FDG PET/CT scans of 63 patients with suspected recurrent RCC after nephrectomy, confirmed with histological examination and/or clinical follow-up and conventional imaging modalities (24). The results of the 103 FDG PET/CT scans were 63 true positive studies, 30 true negative studies, 7 false negative studies, and 3 false positive studies. 109 lesions were detected by FDG PET/CT in the 63 true positive scans. FDG PET/CT was demonstrated to have 90% sensitivity, 91% specificity, and 90% accuracy in the study. Bertagna et al. retrospectively evaluated 68 patients with renal carcinoma who had postoperative FDG PET/CT following partial or radical nephrectomy (25). FDG PET/CT was reported to have 82% sensitivity, 100% specificity, 100% positive predictive value, 66.7% negative predictive value, and 86.6% accuracy. In another study reported by Fuccio et al., the usefulness of FDG PET/CT was assessed in the restaging of 69 RCC patients with clinical or radiological suspicion of metastases after nephrectomy (26). Validation of FDG PET/CT results was established by biopsy, other imaging modalities, and/or clinical and radiological follow-up of 12 months. Forty patients had true positive, 2 patients false positive, 23 patients true negative, and 4 patients false negative. Sensitivity, specificity, accuracy, positive predictive value, and negative predictive value were 90, 92, 91, 95, and 85%, respectively. On a lesion basis, FDG PET/CT detected 114 areas of abnormal uptake in 42 positive patients of which 112 resulted to be true positive.

In another large series study, Win and Aparici retrospectively reviewed the FDG PET/CT studies in 315 RCC patients with biopsy results (27). FDG PET/CT studies exhibited 100% sensitivity and 100% specificity in detecting all metastatic lesions of RCC, the smallest of which detected was a 7 mm lymph node. Therefore, the authors recommended the use of FDG PET/CT in routine standard protocols for RCC.

18F-fluoro-2-deoxy-2-d-glucose (FDG) PET/CT is a valuable tool both in guiding management and treatment in patients with RCC, as well as in predicting survival and progression. A more recent study confirmed the clinical role of FDG PET/CT in the restaging of RCC in a large group of patients (28). For recurrent and/or metastatic lesions in 104 patients, FDG PET/CT demonstrated sensitivity and specificity of 74 and 80%, respectively. FDG PET/CT findings affected management therapies in 45/104 cases (43%). In looking at overall survival (OS), positive FDG PET/CT associated with lower cumulative survival rates cover a 5-year period compared with that of negative FDG PET/CT. Likewise, a positive FDG PET/CT was associated with a lower 3-year progression-free survival (PFS) rate and was associated with high risk of progression, alone or in combination with disease stage or nuclear grading.

**Figure 1:**
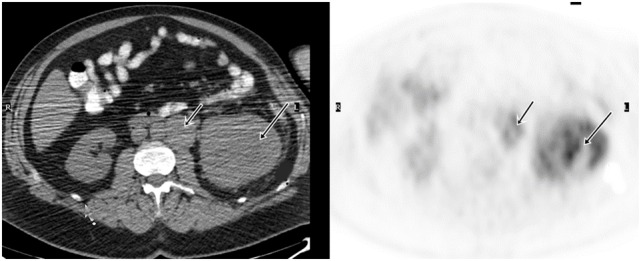
**Demonstration of primary RCC and tumor thrombosis on FDG PET/CT**. A 53-year-old man had a large left renal mass seen on the CT. FDG PET/CT showed increased, heterogeneous uptake of the mass in the left kidney. There was also tumor thrombosis in the renal vein, evidenced by FDG avid intraluminal lesion.

In patients with underlying primary malignancy, there is a high incidence of thrombosis, which can develop from venous thromboembolism (VTE) or more rarely, tumor thrombus. VTE is a common occurrence in cancer, managed with anticoagulant therapy, while tumor thrombosis requires aggressive multimodality therapeutics. Tumor thrombosis most commonly develops in solid tumors, such as RCC and hepatocellular carcinoma, adjacent to large veins as an extension of the malignancy and/or tumor infiltration (29). Sharma et al. conducted a retrospective review of FDG PET/CT scans in patients who had FDG-avid thrombosis (30). FDG PET/CT results were confirmed with clinical follow-up, structural imaging, and histopathology when available. On the basis of structural imaging and clinical follow-up, 10 patients had benign and 14 patients had tumor thrombosis. The most common site of thrombosis was the inferior vena cava. The mean SUV_max_ was 3.2 in the benign thrombosis group and 6.0 in the tumor thrombosis group. The difference in SUV_max_ was significant. In Ravina’s series (31), out of 21 tumor thrombosis cases incidentally detected by FDG PET/CT, 6 were from RCCs. Ferda et al. also reported that FDG PET/CT successfully detected all 7 cases with tumor invasion into the inferior vena cava of 60 patients with RCC (19). The results showed that SUV and the pattern of FDG uptake are helpful for differentiating benign or bland emboli from tumor thrombosis in RCC patients, which is essential for management of patients (Figure 1).

Bone lesions associated with RCC are typically osteolytic. Traditional bone scintigraphy with Tc-99m methylene diphosphonate has limited sensitivity compared with FDG PET/CT, which has a higher sensitivity and a better accuracy in detecting bone metastases in patients with RCC. Wu et al. compared FDG PET with bone scan in 18 patients with biopsy-proven RCC and suspected bone metastases confirmed by histopathology or clinical follow-up of at least 1 year and conventional imaging or FDG PET/bone scans (32). Fifty-two bone lesions, 40 metastatic, and 12 benign, were found on either FDG-PET or bone scan. FDG PET accurately diagnosed all 40 metastatic and 12 benign bone lesions. In comparison, only 31 metastatic bone lesions were accurately detected by bone scan. FDG PET had 100% diagnostic sensitivity and 100% accuracy while that of bone scan were 77.5 and 59.6%, respectively.

**Figure 2:**
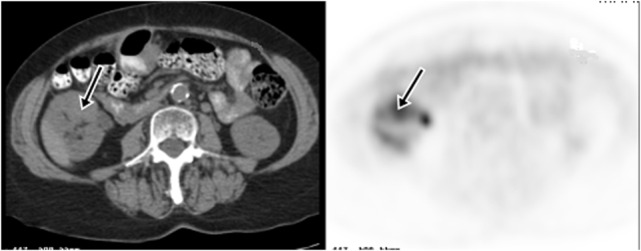
**Demonstration of RCC recurrence on FDG PET/CT**. A 66-year-old woman had right partial nephrectomy for RCC. Two years later, a diagnostic CT showed a new mass in the anterior midpole of the right kidney, which was FDG avid on PET imaging. Subsequent nephrectomy confirmed recurrence of RCC.

**Figure 3:**
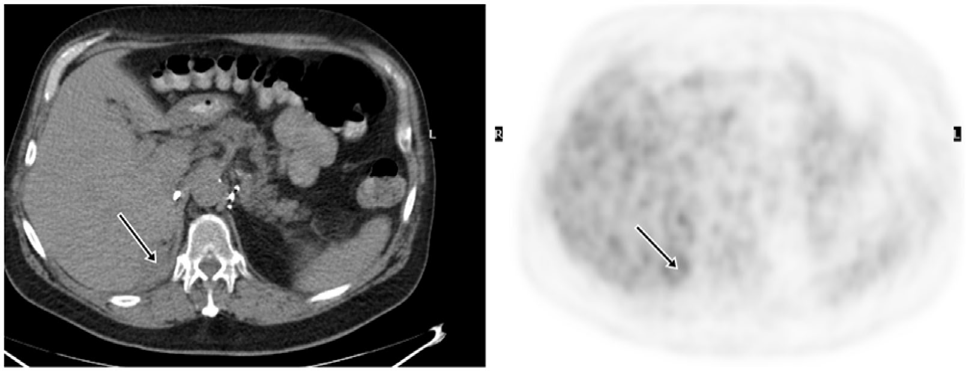
**Demonstration of RCC recurrence on FDG PET/CT**. A 68-year-old man had right radical nephrectomy for RCC. FDG PET/CT was obtained for surveillance 5 years later, which showed a 2.0 cm density with moderate uptake in the surgical bed and was suspicious for recurrence. Surgical pathology revealed recurrent malignancy.

**Figure 4:**
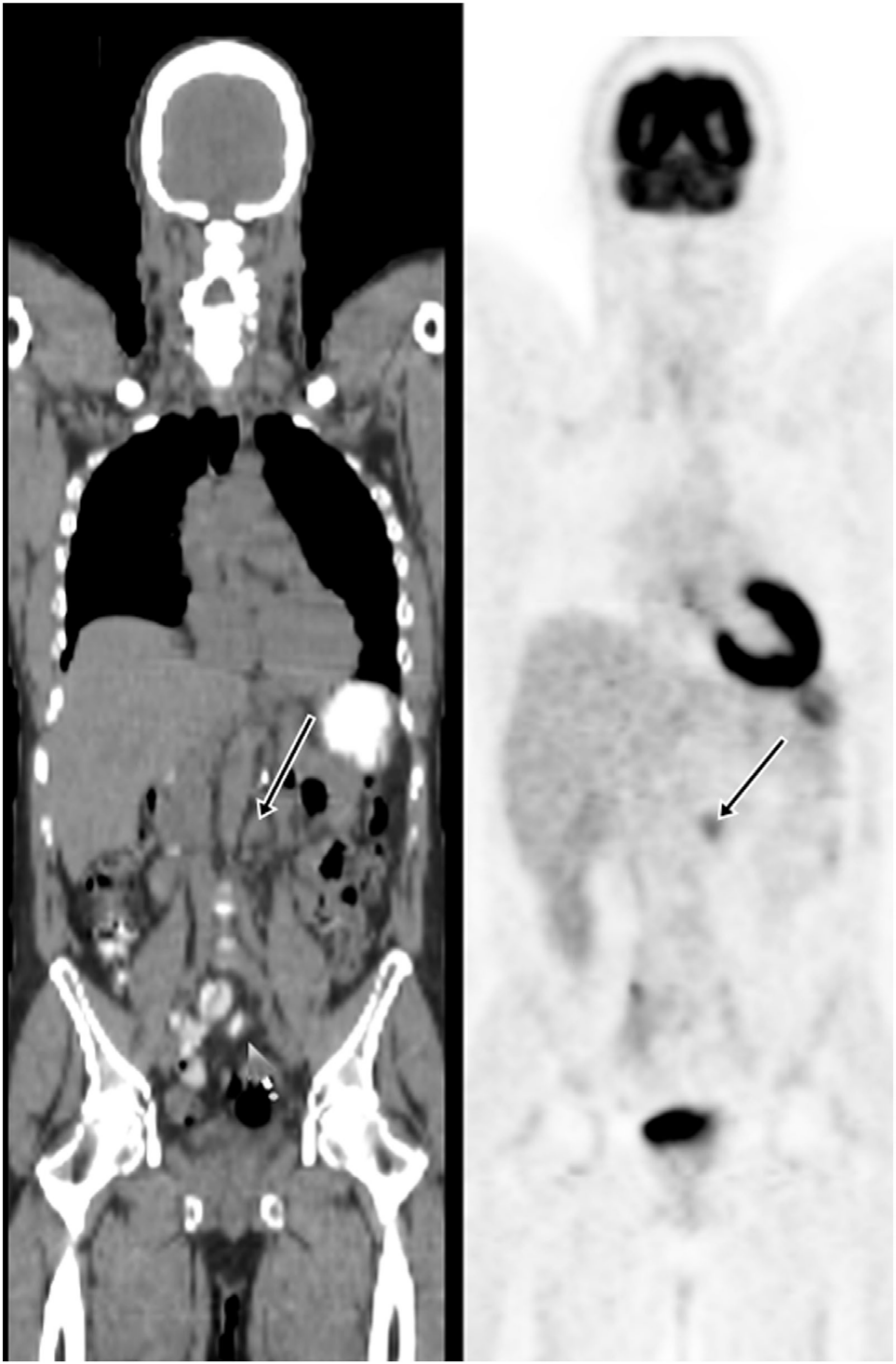
**Demonstration of metastatic lymph node on FDG PET/CT**. A 57-year-old man had the left nephrectomy for RCC 5 years ago. A restaging FDG PET/CT showed a 1.5 cm left para-aortic node with moderate uptake. Subsequent node dissection confirmed metastasis.

18F-fluoro-2-deoxy-2-d-glucose (FDG) PET/CT can provide useful information and has a strong clinical role in the management of recurrent and metastatic RCC (Figures 2–4). In a 58-patient series reported by Rodriguez Martinez de Llano et al. (33), FDG PET/CT had the clinical impact in 25 cases (43%) and no impact in only 10 studies (17.2%). In more recently reported large series by Alongi et al. (28), FDG PET/CT findings influenced therapeutic management in 45/104 cases (43%), treatment was switched from palliative to salvage in 12 patients, and new chemotherapy or immunotherapy was initiated in 24 patients.

Compared to conventional imaging modalities, FDG PET/CT has the advantage in detection of early metastatic disease and identification of musculoskeletal metastases, which are difficult to assess on CT and MRI. Bertagna et al. reported that histologically confirmed bone metastases were revealed at FDG PET/CT in the presence of negative diagnostic CT in 3 out of 27 cases (25). Park et al. compared FDG PET/CT to conventional imaging modalities for restaging 63 patients with RCC who have a high risk of local recurrence or distant metastasis (34). FDG PET/CT accurately classified the presence of a recurrence or metastasis in 56 (89%) patients. FDG PET/CT had 89.5% sensitivity, 83.3% specificity, 77.3% positive predictive value, 92.6% negative predictive value, and 85.7% accuracy in detecting recurrence or metastasis, which were similar to the results with conventional methods. Since FDG PET/CT is versatile and examines all organ systems with high accuracy in one procedure, and with no need for contrast agents, it might replace conventional methods for restaging RCC. Additionally, FDG PET/CT has a unique value in the prediction of survival and risk of progression in patients with recurrent or metastatic RCC (28).

However, FDG is not specific for malignant neoplasm. Increased uptake can be seen in many benign tumors and non-neoplastic processes. On FDG PET/CT for RCC, the false positive results are often due to concomitant inflammatory/infectious disease (9, 28), postoperative scar (26), postradiation inflammation, etc. The most common reason of a false negative FDG PET/CT finding is the small size of lesion and limited spatial resolution of PET scanner (26, 28). In RCC, another potential source of false negative result may be due to close proximity of the lesion to the urinary tract where there is physiologic urine activity (26).

## FDG PET/CT for Monitoring Therapeutic Response to Tyrosine Kinase Inhibitor

Adjuvant therapy remains a poor treatment alternative for advanced RCC. RCC is resistant to both conventional cytotoxic chemotherapy and radiation therapy, which carry a significant toxicity burden. However, a variety of targeted therapies including tyrosine kinase inhibitors (TKIs) have showed promising efficacy in advanced or metastatic RCC, with satisfactory results on PFS and quality of life (35, 36). TKIs, such as sunitinib and sorafenib, are antiangiogenic and can effectively inhibit tumor proliferation.

Although tumor size measurements with the response evaluation criteria in solid tumors (RECIST) criteria have been used for monitoring response to chemotherapy, there is often little change in size of the lesions, and some metastases even increase in size while the drug is prolonging survival (37). In the recent years, FDG PET/CT has been increasingly used to assess the therapeutic efficacy of TKIs in patients with metastatic RCC. According to Caldarella’s systematic review of seven published studies, a good correlation was found between partial metabolic response and PFS and/or OS, with the highest survival rates in patients showing the greatest post-therapeutic reduction in SUV_max_ (38). In contrary, increase on FDG uptake was associated with lower OS (39). Pooled studies showed that FDG PET/CT had a high predictive value in the evaluation of response to SKI treatment in both skeletal and soft tissue lesions of metastatic RCC although there was heterogeneity of available data (38).

Some studies compared the values of FDG PET/CT and RECIST in predicting PFS and OS of patients treated with SKIs for metastatic RCC. Lyrdal et al. reported that FDG PET/CT was more useful than RECIST criteria, especially for the evaluation of skeletal lesions (40), as RECIST is limited to soft tissue lesions.

Kakizoe et al. reported that the decreased ratio of FDG accumulation of RCC lesions, as assessed 1 month following initiation of TKI treatment by FDG PET/CT, was not influenced by the site of RCC metastasis (41). The study suggests that TKIs can be used in the treatment of advanced RCC regardless of the metastatic site, and that FDG PET/CT is a useful method of surveillance to monitor therapeutic response in all lesions.

## Conclusion

Although the usefulness of FDG PET/CT in primary RCC remains unclear, and FDG PET/CT is not currently recommended for the diagnosis and staging of RCC based on updated national and international guidelines (42–44), it can effectively be used for postoperative surveillance and restaging as an adjunct when conventional imaging is not conclusive, as early diagnosis of recurrent/metastatic disease can drastically affect therapeutic decision and alter outcomes of patients (45). FDG uptake is helpful for differentiating benign or bland emboli from tumor thrombosis in RCC patients. FDG PET/CT has a higher sensitivity and accuracy in detecting bone metastases in patients with RCC than that of bone scan. Pretreatment SUV_max_ assessed by FDG PET/CT can provide helpful information for clinical decision-making as it can serve as a useful prognostic marker for patients with advanced RCC. High SUV_max_ in patients with primary RCC is suggested with correlate with a high likelihood of metastasis, and FDG accumulation may be useful in estimating patient’s survival. In monitoring the efficacy of TKI treatment for advanced RCC, FDG PET/CT has been increasingly used to assess the therapeutic efficacy, and change of FDG uptake is a powerful index for evaluating the biological response to TKI.

## Author Contributions

The author confirms being the sole contributor of this work and approved it for publication.

## Conflict of Interest Statement

The author declares that the research was conducted in the absence of any commercial or financial relationships that could be construed as a potential conflict of interest.
